# RNA sequencing reveals dynamic expression of spleen lncRNAs and mRNAs in Beagle dogs infected by *Toxocara canis*

**DOI:** 10.1186/s13071-022-05380-x

**Published:** 2022-08-04

**Authors:** Yue Xu, Wen-Bin Zheng, Hao-Yu Li, Lang Cai, Yang Zou, Shi-Chen Xie, Xing-Quan Zhu, Hany M. Elsheikha

**Affiliations:** 1 Laboratory of Parasitic Diseases, College of Veterinary Medicine, Shanxi Agricultural University, Taigu, Shanxi Province 030801 People’s Republic of China; 2 State Key Laboratory of Veterinary Etiological Biology, Key Laboratory of Veterinary Parasitology of Gansu Province, Lanzhou Veterinary Research Institute, Chinese Academy of Agricultural Sciences, Lanzhou, Gansu Province 730046 People’s Republic of China; 3 Key Laboratory of Veterinary Public Health of Higher Education of Yunnan Province, College of Veterinary Medicine, Yunnan Agricultural University, Kunming, Yunnan Province 650201 People’s Republic of China; 4 School of Veterinary Medicine and Science, Faculty of Medicine and Health Sciences, University of Nottingham, Sutton Bonington Campus, Loughborough, LE12 5RD UK

**Keywords:** *Toxocara canis*, lncRNA, mRNA, Transcriptomics, Beagle dog, Spleen

## Abstract

**Background:**

*Toxocara canis* is a cosmopolitan parasite with a significant adverse impact on the health of humans and animals. The spleen is a major immune organ that plays essential roles in protecting the host against various infections. However, its role in *T. canis* infection has not received much attention.

**Methods:**

We performed sequencing-based transcriptome profiling of long noncoding RNA (lncRNA) and messenger RNA (mRNA) expression in the spleen of Beagle puppies at 24 h post-infection (hpi), 96 hpi and 36 days post-infection (dpi). Deep sequencing of RNAs isolated from the spleen of six puppies (three infected and three control) at each time point after infection was conducted.

**Results:**

Our analysis revealed 614 differentially expressed (DE) lncRNAs and 262 DEmRNAs at 24 hpi; 726 DElncRNAs and 878 DEmRNAs at 96 hpi; and 686 DElncRNAs and 504 DEmRNAs at 36 dpi. Of those, 35 DElncRNA transcripts and 11 DEmRNAs were detected at all three time points post-infection. Many DE genes were enriched in immune response, such as *ifit1*, *ifit2* and *rorc*. Kyoto Encyclopedia of Genes and Genomes enrichment analysis revealed that some genes (e.g. *prkx* and *tnfrsf11a*) were involved in the T cell receptor signaling pathway, calcium signaling pathway, Ras signaling pathway and NF-κB signaling pathway.

**Conclusions:**

The findings of this study show marked alterations in the expression profiles of spleen lncRNAs and mRNAs, with possible implications in the pathophysiology of toxocariasis.

**Graphical Abstract:**

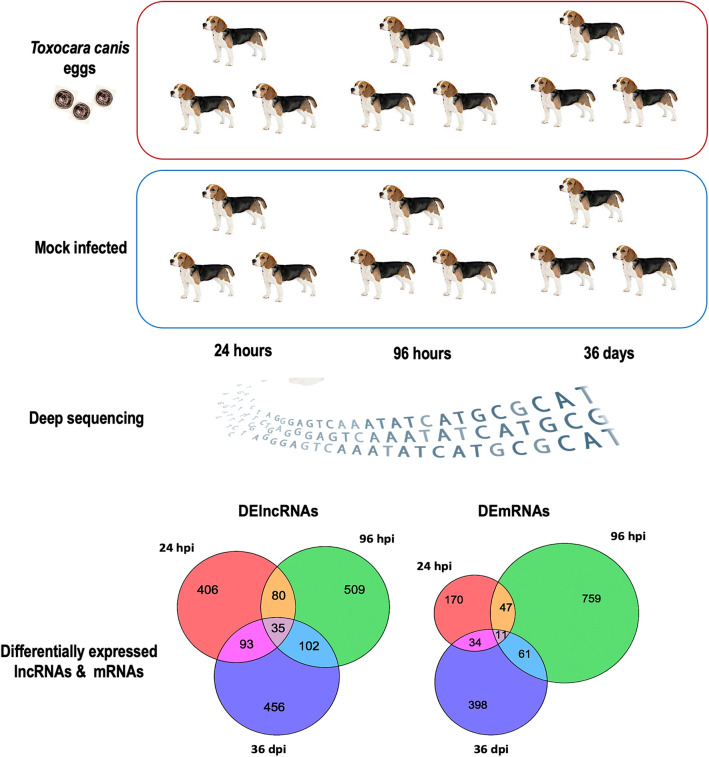

**Supplementary Information:**

The online version contains supplementary material available at 10.1186/s13071-022-05380-x.

## Background

Toxocariasis, a worldwide zoonotic helminth infection, is caused by *Toxocara canis* and, to a lesser extent, *Toxocara cati* [1]. *Toxocara canis* is a common parasitic nematode of dogs and other canine species [2]. Infected dogs excrete *T. canis* eggs into the environment where they pose a significant public health problem in many countries [3, 4]. Infection in humans can lead to visceral larva migrans (VLM), ocular larva migrans (OLM) or neurotoxocariasis (NT) [5]. To date, many aspects of the pathophysiology of toxocariasis remain unclear, and there is no information available on gene expression changes in the dog’s spleen in response to *T. canis* infection.

As the largest lymphoid organ in the body, the spleen is an important site for innate and adaptive immune responses [6, 7]. Despite its involvement in various immunomodulatory functions, the role of the spleen in *T. canis* infection is unknown. Understanding changes in gene expression and regulatory pathways of dog splenic tissue during *T. canis* infection can provide new avenues for understanding the pathogenesis of toxocariasis. The development of next-generation sequencing and other high-throughput ‘omics’ technologies have provided an unprecedented opportunity to investigate the molecular features and pathogenesis of *T. canis* infection [8, 9].

Previous research revealed significant alterations in long noncoding RNA (lncRNA) and messenger RNA (mRNA) expression profiles in the lung and bone marrow of Beagle puppies and identified a dynamic balance between pro-inflammatory and anti-inflammatory responses that was relevant to the development and migration of *T. canis* in the definitive canine host [10, 11]. LncRNAs are a class of non-coding transcripts defined by a threshold of > 200 nucleotides that reside within or between coding genes [12]. LncRNAs regulate various biological and pathological processes at the epigenetic and transcriptional levels [13–15] and can influence host immune response to pathogens [16, 17]. To our knowledge, comprehensive lncRNA and mRNA expression profiling in the spleen of dogs infected by *T. canis* has not been performed to date.

In the present study, we profiled the expression levels of lncRNAs and mRNAs in the spleen of Beagle puppies at different time points after *T. canis* infection using RNA sequencing (RNA-seq). The transcriptional profiling data suggest important roles for lncRNAs and mRNAs in the pathogenesis of toxocariasis.

## Methods

### Animal infection and sample collection

Eighteen 6-to-7-week-old *Toxocara*-naive Beagle puppies were used in this study. Information on the rearing environment of the puppies, absence of prior infection and detection of experimental *T. canis* infection has been described in previous studies [10, 11, 18]. Briefly, 18 puppies were randomly assigned to three groups, with each group representing a time point of larval development in the host: 24 hours post-infection (hpi), 96 hpi and 36 days post-infection (dpi). Each group of six dogs, included three infected and three control dogs. To induce infection, we orally inoculated three puppies in each group with 300 embryonated *T. canis* eggs in 1 ml of sterile saline; the control puppies in each group were inoculated with 1 ml of saline, without eggs. Puppies were euthanized by intracardiac injection of potassium chloride (KCl) solution, under general anesthesia using Zoletil™ 50 (Virbac, Carros, France). This procedure was performed by a trained veterinarian with knowledge of anesthetic techniques to ensure the suitability of the anesthetic depth prior to administration of KCl. After washing the surface of spleen with saline solution, approximately  2 g of splenic tissue was dissected out of each puppy and stored in liquid nitrogen for extraction of total RNA. None of the puppies exhibited any adverse health effects or died before they were euthanized.

### RNA extraction and sequencing

Total RNA was extracted from the splenic tissue sample of each puppy, followed by removal of genomic DNA using DNaseI (New England BioLabs, Ipswich, MA, USA). Total RNA degradation was monitored in 1% agarose gels, and the integrity of each total RNA was measured using the Bioanalyzer 2100 system (Agilent Technologies Inc., Santa Clara, CA, USA). Those total RNAs with RNA integrity number (RIN) ≥ 8 was selected for sequencing analysis [19]. Construction of the libraries and RNA sequencing were conducted as described previously [10]. Briefly, 3 µg RNA extracted from each spleen was used to generate sequencing libraries using NEBNext® UltraTM RNA Library Prep Kit (New England BioLabs), and index codes were added per each RNA sample. Complementary DNA (cDNA) fragments with a length of 150–200 bp were selected. PCR assays were conducted using universal PCR primers, Phusion High-Fidelity DNA polymerase and Index (X) Primer (both New England BioLabs). The quality of the library was measured by using the Agilent Bioanalyzer 2100 system (Agilent Technologies Inc.). The clustering of the index-coded samples was conducted on a cBot Cluster Generation System using TruSeq PE Cluster Kit v3-cBot-HS (Illumina Inc., San Diego, CA, USA). The 150-bp paired end reads were sequenced on an Illumina Hiseq 2500 instrument for each library.

### Identification of lncRNAs and differentially expressed lncRNAs and mRNAs

Clean data were obtained by removing the raw reads containing poly-N, adapter and low-quality reads. GC content and the Q20 and Q30 of the clean data were determined. Genome and annotation files of *Canis lupus familiaris* were downloaded from the Ensembl database (CanFam3.1; https://www.ensembl.org/index.html). The Index of the reference genome was built using STAR software, and paired-end clean reads were aligned to the reference genome using STAR (v2.5.1b) [20]. The reads were spliced into transcripts using StringTie software based on the results compared to the genome [21], and each transcript generated a stream neural network with a maximum stream algorithm to evaluate its expression level for quantification. The transcripts and genes obtained from the splicing of each sample were merged using Cuffmerge software and compared with known databases using Cuffcompare software to obtain known_lncRNA and known_mRNA. The known transcripts were then filtered out, and the remaining transcripts were used to analyze the coding potential and to obtain novel_lncRNAs and novel_mRNAs using the CPC2, Pfam and CNCI [22]. HTSeq v0.6.0 was used to count the numbers of reads mapped to each gene. The FPKM (fragments per kilobase million) of each gene, based on the length and reads count of the gene, was used to determine the expression levels. The transcript level for lncRNAs with *P*-value < 0.05 and log2 (fold change) ≥ 2, and the gene level for mRNAs with *P*-value < 0.05 and log2 (fold change) ≥ 0.5 indicate differential expression, using the DESeq2 (version 1.10.1). The numbers of differentially expressed lncRNA transcripts (DElncRNAs) and differentially expressed mRNAs (DEmRNAs) were analyzed at the three infection stages using GraphPad Prism software v7.0.0 (GraphPad Software, San Diego, CA, USA).

### Gene Ontology annotation and Kyoto Encyclopedia of Genes and Genomes enrichment analysis

The target genes of lncRNAs were predicted based on positional relationship (co-location) of lncRNAs with the nearby protein-coding genes [23]. The regulatory networks of interacting DElncRNAs-DEmRNAs at 24 hpi, 96 hpi and 36 dpi were constructed and visualized using Cytoscape v3.9.0. GOseq R software and KOBAS (KEGG Orthology-Based Annotation System) were used to perform Gene Ontology (GO) and Kyoto Encyclopedia of Genes and Genomes (KEGG) analyses for the potential target genes of DElncRNA and DEmRNAs, respectively [24, 25]. GO terms with *P*-value < 0.05 were deemed significantly enriched using the t-test.

### Real-time quantitative PCR

The expression of 30 transcripts (15 DEmRNAs and 15 DElncRNAs) was analyzed by quantitative real-time PCR (qRT-PCR) on a LightCycler 480 PCR platform (Roche, Basel, Switzerland) to verify the results of RNA-seq at the three infection stages. The amplification program, kits and melting curve analysis of qRT-PCR were performed strictly as previously described [10], and GraphPad Prism v7.0.0 software was used to depict the validation results. *L13A* (ribosomal protein L13A), a house-keeping gene, was used as an internal control for normalizing the level of mRNAs and lncRNAs. The selected transcripts and primers used are listed in the Additional file 1: Table S1.

## Results

### Characteristic features of RNA-seq data

**Fig. 1:**
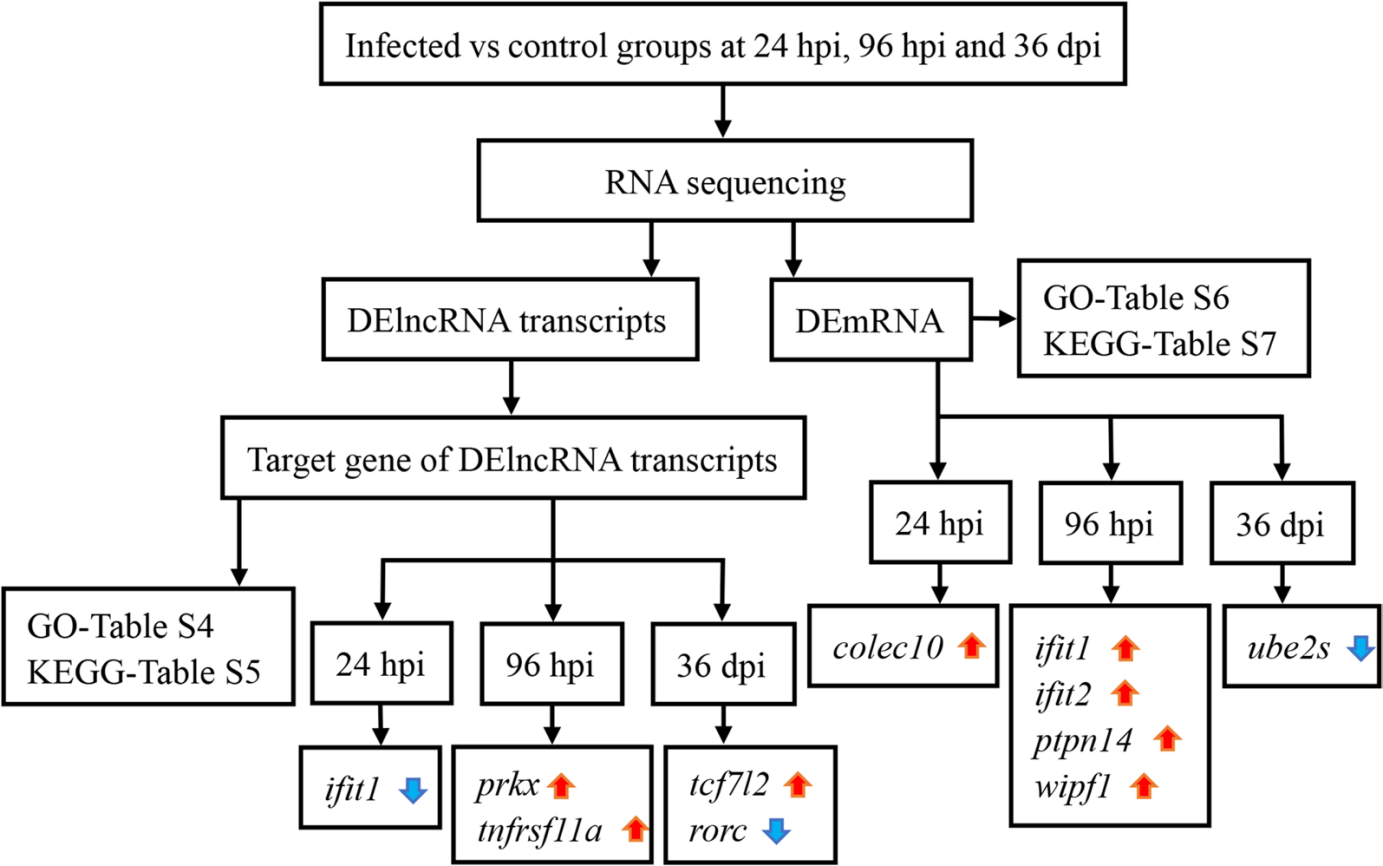
Schematic summary of the study workflow. See Abbreviation list for explanations

A total of 1,909,813,998 raw reads and 1,892,781,894 clean reads were obtained from spleen samples of 18 puppies, with an average of 15.77 Gb clean reads per sample. The mean values of GC and Q30 in the raw reads were 46.86% and 94.17%, respectively, showing the good quality of the RNA-seq data. Additionally, 95.68% clean reads could be mapped to the reference genome, and 12,283 annotated lncRNA transcripts, 45,036 mRNA transcripts and 10,824 novel lncRNA transcripts and 462 novel mRNA transcripts were identified. The schematic workflow of data processing is shown in Fig. 1.

### Patterns of DElncRNAs and DEmRNAs

**Fig. 2:**
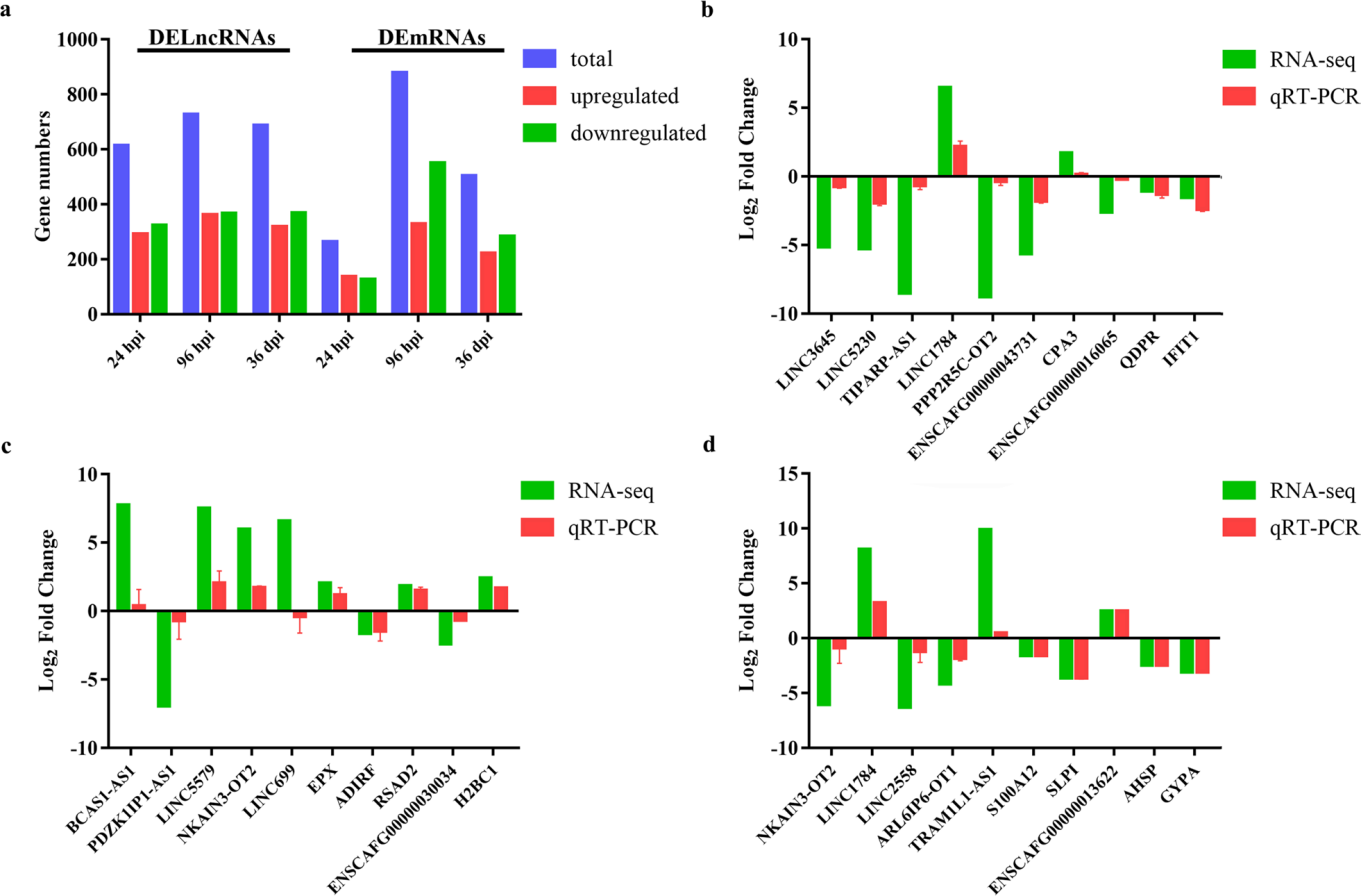
Differential expression of lncRNA transcripts and mRNAs in the spleen of Beagle puppies infected by *Toxocara canis* at 24 hpi, 96 hpi, and 36 dpi. **a** Number of DElncRNAs transcripts and DEmRNAs at three infection stages. Blue, red and green colors represent total, upregulated or downregulated total transcripts and genes, respectively. **b**–**d** Verification of the expression of DElncRNAs and DEmRNAs using qRT-PCR at 24 hpi (**b**), 96 hpi (**c**) and 36 dpi (**c**). * Y*-axis denotes the log2 fold change, and* X*-axis shows the analyzed DElncRNAs and DEmRNAs. Error bars represent the standard deviation based on three replicates. DElncRNAs, Differentially expressed lncRNA transcripts; DEmRNAs, differentially expressed mRNAs; dpi, days post-infection; hpi, hours post-infection; qRT-PCR, quantitative real-time PCR; RNA-seq, RNA sequencing

**Fig. 3:**
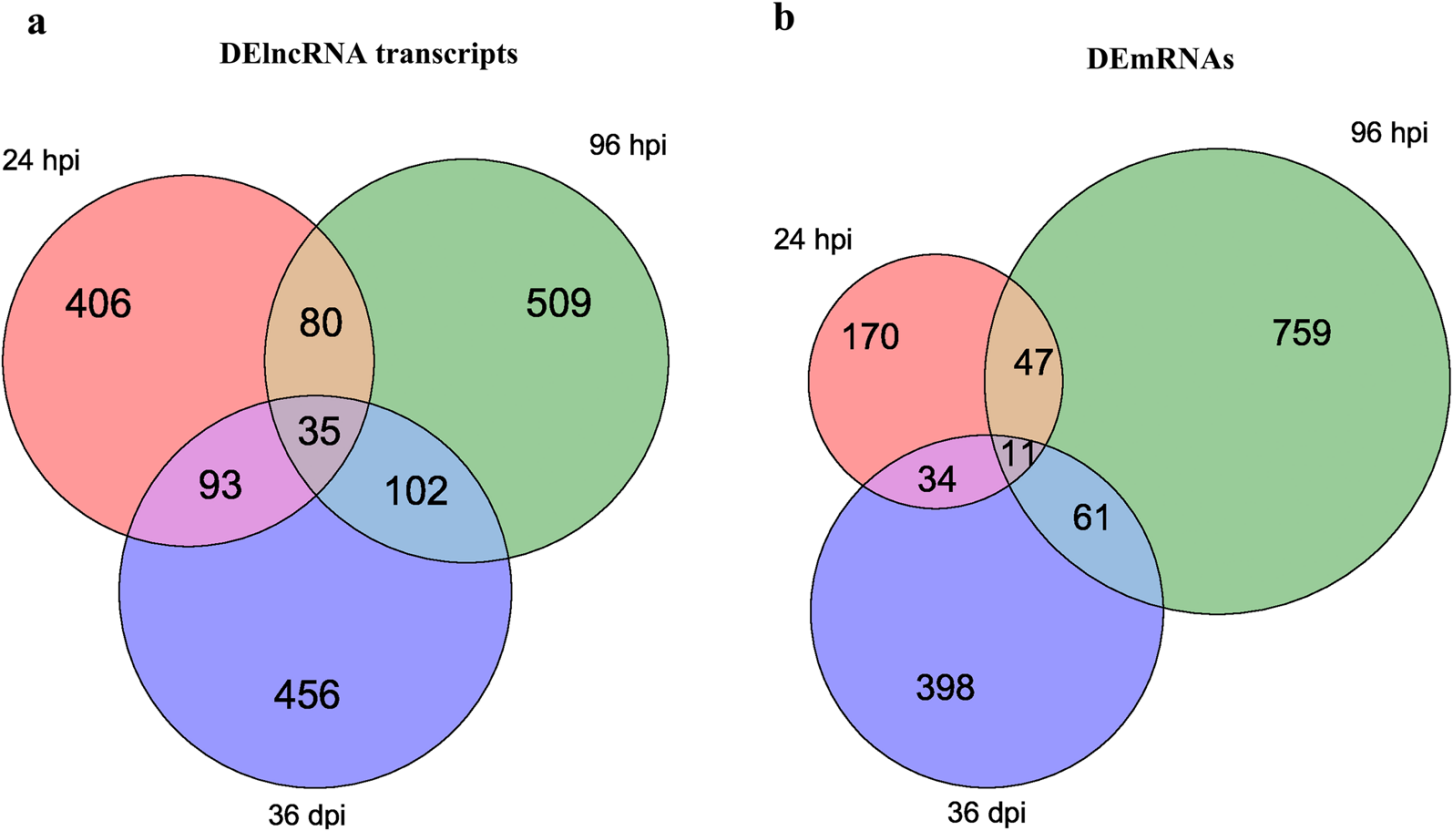
Venn diagrams showing the common and unique DElncRNA transcripts (**a**) and DEmRNAs (**b**) in the spleen of Beagle puppies infected by *Toxocara canis* at 24 h hpi, 96 hpi and 36 dpi

At 24 hpi, 614 DElncRNAs and 262 DEmRNAs were identified. At 96 hpi, 726 DElncRNAs and 878 DEmRNAs were identified. At 36 dpi, 686 DElncRNAs and 504 DEmRNAs were identified (Fig. 2a; Additional file 2: Table S2). In addition, 35 DElncRNAs and 11 DEmRNAs were commonly identified at all three time points post-infection (Fig. 3), suggesting their essential roles in the pathogenesis of *T. canis* infection.

### qRT-PCR confirms RNA-seq results

For a subset of the DE transcripts, qRT-PCR was used as an independent validation method to examine the differences in expression between results obtained by RNA-seq and those obtained by qRT-PCR. The results of the subset analysis showed that the levels of expression of 15 DEmRNAs and 15 DElncRNAs obtained by qRT-PCR were comparable to the expression levels detected by RNA-seq (Fig. 2b–d).

### Target gene prediction and functional analysis of lncRNAs

A total of 16,241 mRNAs were found in the 100-kb fragment up- and downstream of the 17,142 lncRNA transcripts. At 24 hpi, 20 DEmRNAs (e.g. *ifit1* [Interferon-induced protein with tetratricopeptide repeats 1] and *colec10* [Collectin subfamily member 10]) were identified up- and downstream of 20 DElncRNA transcripts. At 96 hpi, 81 DEmRNAs (e.g. *ifit2* and *colec10*) were identified up- and down-stream of 90 DElncRNA transcripts. At 36 dpi, 47 DEmRNAs (e.g. *ube2s* [Ubiquitin-conjugating enzyme E2S]and *tcf7l2* [transcription factor 7 like 12]) were identified up- and downstream of 52 DElncRNA transcripts (Additional file 3: Figure S1; Additional file 4: Table S3).

**Fig. 4:**
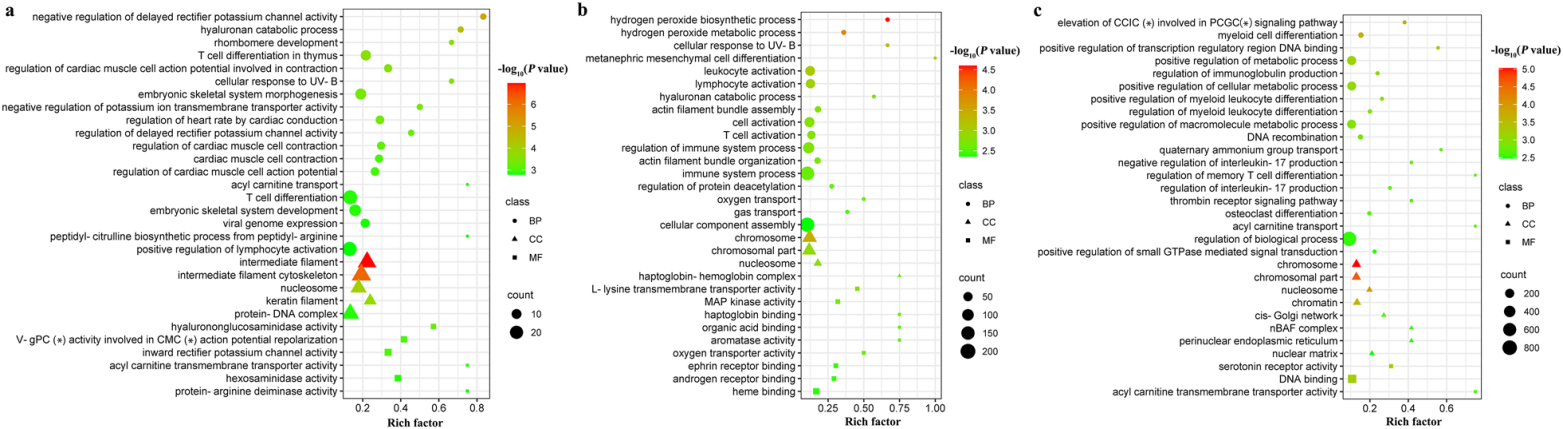
Scatter plots of the top 30 enriched Gene Ontology (GO) terms (including biological process [BP], cellular component [CC] and molecular function [MF] categories) of the DElncRNA transcript target genes at 24 h hpi (**a**), 96 hpi (**b**) and 36 dpi (**c**). The* X*-axis label represents the rich factor; the* Y*-axis label shows the GO terms. The rich factor reflects the proportion of target mRNAs in a given GO term. The color of the dots represents the enrichment score [− log_10_ (*P*-value)], where red indicates high enrichment and green indicates low enrichment. Dot size represents the number of mRNAs in the corresponding GO term (larger dots indicate larger numbers of target mRNAs). CCIC, Cytosolic calcium ion concentration; CMC, cardiac muscle cell; PCGC, phospholipase C-activating G-protein coupled; V-Gpc, voltage-gated potassium channel

GO and KEGG enrichment analyses were conducted on the predicted target genes of DElncRNAs. A total of 408, 439 and 435 GO terms were significantly enriched at 24 hpi, 96 hpi and 36 dpi, respectively (Additional file 5: Table S4). The top 30 significantly enrichment GO terms of the predicted target genes of DElncRNAs are shown in Fig. 4. At 24 hpi, 10 pathways were significantly enriched, including, for example, RNA transport, base excision repair and glycerophospholipid metabolism. At 96 hpi, 25 pathways were significantly enriched, including, for example, base excision repair, asthma and graft-versus-voltage metabolism and asthma and graft-versus-host disease. At 36 dpi, 19 pathways were significantly enriched, including, for example, base excision repair, asthma, allograft rejection, graft-versus-host disease and malaria (Additional file 6: Figure S2; Additional file 7: Table S5).

### GO and KEGG enrichment analysis of DEmRNAs

The functional classification and enrichment analysis of DEmRNAs showed that 134 DEmRNAs were significantly enriched in 221 GO terms at 24 hpi. Of those, 127 belonged to the biological process (BP) category (e.g. inflammatory response, response to histamine, negative regulation of inflammation, response to histamine, and glutamate receptor signaling pathway), 23 belonged to the cellular component (CC) category (e.g. mitochondrial proton-transporting ATP synthase complex, mitochondrial proton-transporting ATP synthase-stator stalk, proton-transporting ATP synthase-stator stalk, alpha-amino-3-hydroxy-5-methyl-4-isoxazolepropionic acid selective glutamate receptor complex and proton-transporting ATP synthase complex), and 71 belonged to the molecular function (MF) category (e.g. catalase activity, peroxidase activity, oxidoreductase activity-acting on peroxide as acceptor, cofactor binding and sulfotransferase activity). At 96 hpi, 729 DEmRNAs were significantly enriched in 494 GO terms; of these, 308 belonged to the BP category (e.g. translation, cytoplasmic translation, negative regulation of viral genome replication, positive regulation of acute inflammatory response, and regulation of chronic inflammatory response), 88 belonged to the CC category (e.g. ribosome, cytosolic ribosome, ribosomal subunit, ribonucleoprotein complex and cytosolic part), and 98 belonged to the MF category (e.g. structural constituent of ribosome, structural molecule activity, RNA binding, ribosomal RNA binding and protein tag). At 36 dpi, 350 DEmRNAs were significantly enriched in 416 GO terms. Of these, 266 belonged to the BP category (e.g. nucleosome assembly, antimicrobial humoral response, inflammatory response to antigenic stimulus, production of molecular mediator involved in inflammatory response, and cytokine production involved in inflammatory response), 36 belonged to the CC category (e.g. nucleosome, protein-DNA complex, extracellular region, extracellular space and extracellular region part) and 114 belonged to the MF category (e.g. protein heterodimerization activity, peroxidase activity, oxidoreductase activity-acting on peroxide as acceptor, antioxidant activity and RAGE receptor binding) (Additional file 8: Table S6). The top 30 significantly enrichment GO terms are shown in Additional file 9: Figure S3.

**Fig. 5:**
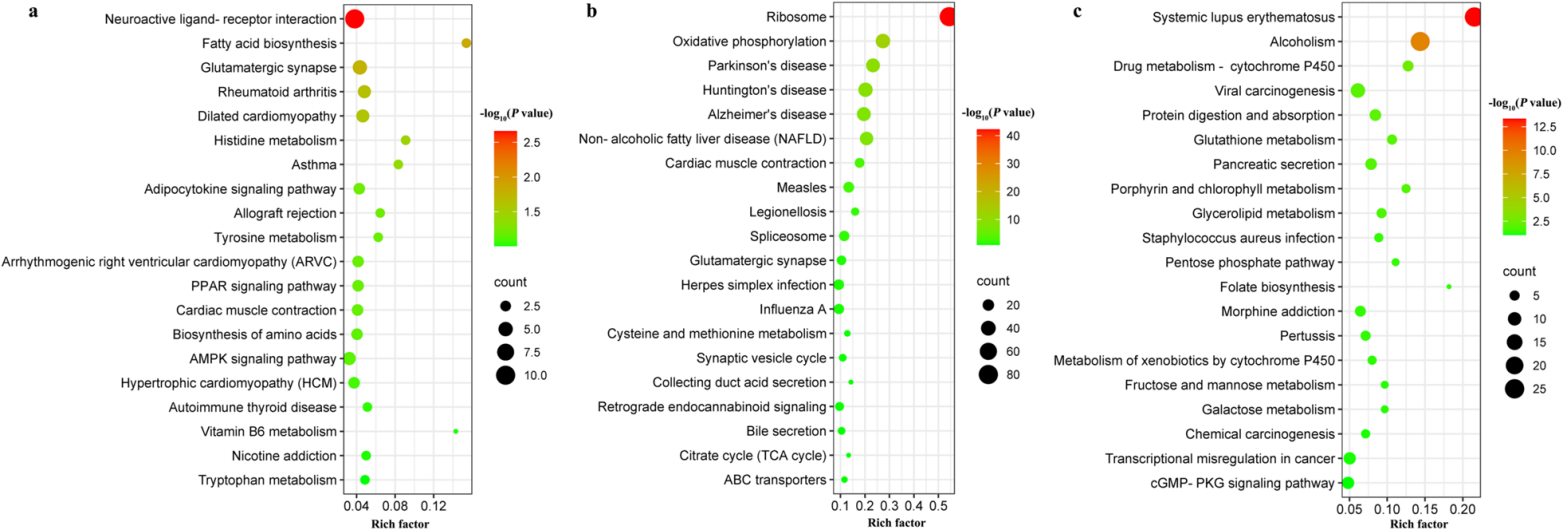
Scatter plots of the top 20 enriched Kyoto Encyclopedia of Genes and Genomes (KEGG) pathways of the DEmRNAs at 24 hpi (**a**), 96 hpi (**b**) and 36 dpi (**c**) in the spleen of Beagle puppies. The* X*-axis label represents the rich factor, and the* Y*-axis label shows the KEGG pathway name. The rich factor reflects the proportion of DEmRNAs in a given pathway. The color of the dots represents the enrichment score [− log_10_ (*P*-value)], where red indicates high enrichment, and green indicates low enrichment. Dot size represents the number of DEmRNAs in the corresponding KEGG pathways (larger dots indicate larger DEmRNA numbers)

KEGG enrichment analysis at 24 hpi showed that 29 DEmRNAs (e.g. *acsl6*, *lep* and *grm1*) were significantly enriched in 7 signaling pathways (e.g. glutamatergic synapse, histidine metabolism and fatty acid biosynthesis). At 96 hpi, 319 DEmRNAs (e.g. *cox5b*, *sdhb* and *ndufa6*) were significantly enriched in 11 signaling pathways (e.g. legionellosis, oxidative phosphorylation and Huntington’s disease). At 36 dpi, 115 DEmRNAs (e.g. *c3*, *h3c13* and *h2ax*) were significantly enriched in 14 signaling pathways, such as pentose phosphate pathway, glycerollipid metabolism and pancreatic secretion signaling pathway (Additional file 10: Table S7). The top 20 most representative pathways in each group are shown in Fig. 5.

## Discussion

This study showed that *T. canis* infection alters the expression of splenic lncRNAs and mRNAs with possible implications for toxocariasis pathophysiology. The spleen transcriptome of Beagle puppies was analyzed by RNA-seq after *T. canis* infection at 24 hpi, 96 hpi and 36 dpi. *Toxocara canis* infection-specific alterations in lncRNAs and mRNAs expression were detected at different time points during *T. canis* infection, suggesting that these changes are directly caused by parasite infection and are not a consequence of infection. In this study, 12,283 annotated lncRNA transcripts and 10,824 novel lncRNA transcripts were identified; of these, 614, 726 and 686 DElncRNA transcripts were identified between control and infected puppies at 24 hpi, 96 hpi and 36 dpi, respectively. Additionally, 35 DElncRNAs were identified at all three post-infection stages. We also identified a total of 45,036 mRNA transcripts and 462 novel mRNA transcripts. A total of 262, 878 and 504 DEmRNAs were identified at 24 hpi, 96 hpi and 36 dpi, respectively. Additionally, 11 DEmRNAs were identified at all infection stages. The detection of more DElncRNAs than DEmRNAs shows the different roles of RNA classes during *T. canis* infection and is consistent with the many functional roles of lncRNAs, in addition to their role of mRNA transcription regulation.

### Expression signatures of splenic lncRNAs

At 24 hpi, 20 DEmRNAs (e.g. *ifit1*) were found up- and downstream of 20 DElncRNA transcripts, forming 22 pairs (Additional file 3: Figure S1a). The results of GO and KEGG analyses showed that 13 predicted target DEmRNAs of DElncRNAs were significantly enriched in 58 GO terms. Of these, *ifit1* was significantly enriched in 14 GO terms, such as host, host cell and immune effector process. The interferon-inducible proteins (IFIT) are not only involved in the interferon (IFN) gamma signaling, which regulates innate immune signaling pathways [26], but they also mediate Toll-like receptor (TLR) signaling [27]. Some members of the TLR family, such as TLR2 and TLR4, are involved in parasitic infections [28]. At 24 hpi, the expression levels of *ifit1* and its corresponding lncRNA (TCONS_00345683) were decreased by 2.83- and 2715.87-fold, respectively. *Toxocara canis* has been shown to have an immunosuppressive effect via the production of immunomodulatory molecules [29]. Thus, the downregulation of TCONS_00345683 and its target gene *ifit1* suggests that *T. canis* downregulates the splenic immune response during the early stage of infection to facilitate larval invasion of host tissue.

At 96 hpi, 81 DEmRNAs (e.g. *prkx* [Protein kinase X-linked] and *tnfrsf11a* [Tumor necrosis factor {TNF} receptor superfamily member 11a]) were identified up- and downstream of 90 DElncRNAs forming 99 pairs (Additional file 3: Figure S1b). A total of 54 predicted target DEmRNAs of DElncRNAs were significantly enriched in 135 GO terms, and 10 predicted target DEmRNAs of DElncRNAs were significantly enriched in six signaling pathways. Protein kinase X-linked (*prkx*) was significantly enriched in the calcium signaling pathway and Ras signaling pathway. The calcium signaling pathway contributes to the generation of Ca^2+^ that controls key processes such as proliferation, secretion and metabolism [30]. Likewise, the Ras signaling pathway is involved in important biological processes, such as proliferation, survival, differentiation and apoptosis [31]. The protein encoded by *prkx* is involved in the maturation of macrophages and granulocytes [32], and it has been shown that macrophages may contribute to parasite containment, inactivation and expulsion in vivo [33, 34]. The level of *prkx* was upregulated 2.29-fold, and the levels of TCONS_00471550 and TCONS_00471551 located near the *prkx* gene were upregulated 884.31- and 1752.19-fold, respectively*,* which may help to limit the migration and development of *T. canis* larvae in the host.

*tnfrsf11a* was significantly enriched in 18 GO terms, such as immune system development and hematopoietic or lymphoid organ development. Also, *tnfrsf11a* was significantly enriched in the NF-κB signaling pathway that regulates the immunity reaction, inflammatory response and cell survival. As a member of the TNF-receptor superfamily, *tnfrsf11a* can interact with multiple TNF receptor-associated factor (TRAF) family proteins involved in the activation of the non-classical pathway of NF-κB [35]. At 96 hpi, *tnfrsf11a* was upregulated 2.08-fold. The levels of TCONS_00001189 located near *tnfrsf11a* was upregulated 681.13-fold and ENSCAFT00000051098 located near *tnfrsf11a* was downregulated 385.99-fold. The concomitant increase in TCONS_00001189 and decrease in ENSCAFT00000051098 may influence *tnfrsf11a* expression to promote immune processes and lymphoid organ development in the infected dogs.

At 36 dpi, 47 DEmRNAs (e.g. *tcf7l2* and *rorc* [Retinoic acid receptor {RAR}-associated orphan receptor C]) were identified up- and downstream of 52 DElncRNAs forming 61 pairs (Additional file 3: Figure S1c). GO annotation analysis showed that 37 predicted target DEmRNAs of DElncRNAs were significantly enriched in 145 GO terms. In addition, five predicted target DEmRNAs of DElncRNAs were significantly enriched in four signaling pathways, of which *tcf7l2* was significantly enriched in 67 GO terms, such as regulation of sequence-specific DNA binding transcription factor activity, regulation of metabolic process and regulation of biological process. *Tcf7l2* is a central factor in cell proliferation or death, metabolism and stress response [36]. Also, *tcf7l2* is a key effector in the formation of *tcf7l2*/*β*-catenin complex, which transcriptionally activates downstream factors in the Wnt pathway [37]. Wnt/*β*-catenin signaling contributes to the development and homeostasis of multiple tissues [38]. Under physiological conditions, accumulated *β*-catenin enters the nucleus and binds to members of the T-cell factor/lymphatic enhancer factor (TCF/LEF) family of high-mobility group transcription factors to induce or repress the transcription of many target genes [39]. The level of *tcf7l2* gene was upregulated 2.21-fold. Also, the levels of TCONS_00258425 and TCONS_00258384 located near *tcf7l2* gene were upregulated 18,596.26- and downregulated 524.27-fold, respectively. The concomitant increase of TCONS_00258425 and decrease of TCONS_00258384 may influence *tcf7l2* to promote *β*-catenin accumulation, thereby activating the metabolism, stress response and cell differentiation/proliferation in the spleen.

Retinoic acid receptor (RAR)-associated orphan receptor C (*rorc*) was significantly enriched in inflammatory bowel disease and circadian rhythm, according to KEGG enrichment analysis, and was significantly enriched in 40 GO terms, such as regulation of gene expression and signaling receptor activity, based on GO annotation analysis. RORC also known as ROR*γ*, plays key roles in differentiation of CD4^+^ T helper cells into Th17 cells [40]. *rorc* also plays regulatory roles in immunity, peripheral circadian rhythms and metabolism of lipids, steroids, xenobiotics and glucose [41]. Since *rorc* encodes transcription factors for Th17 cells, the mRNA levels of these transcription factors are considered to be indicators of T lymphocyte differentiation [42, 43]. T helper cells participate in inflammatory responses, coordinating the adaptive immune responses and host defense against various pathogens [44]. At 36 dpi, the transcriptional level of *rorc* was downregulated 13.840-fold and the level of TCONS_00124090 located near the *rorc* gene was downregulated 1094.66-fold. In contrast, the level of TCONS_00124095 located near the *rorc* gene was upregulated 301.61-fold. TCONS_00124090 and TCONS_00124095 may coordinate the reduction of *rorc* gene expression, resulting in a downregulated splenic immune response via reducing the differentiation of naive CD4^+^ T cells into Th17 cells.

### Expression profiles of splenic mRNAs

At 24 hpi, GO annotation and KEGG analyses showed that 134 DEmRNAs were significantly enriched in 221 GO terms (Additional file 8: Table S6), and 23 DEmRNAs were significantly enriched in seven signaling pathways (Additional file 10: Table S7). *Colec10* was significantly enriched in extracellular space according to GO enrichment analysis. This gene encodes lectin liver 1 (CL-L1) and is associated with the activation of the complement system [45]. In this study, the transcriptional level of *colec10* was upregulated 399.76-fold and the level of TCONS_00074002 located near *colec10* was upregulated 585.34 fold. The upregulation of TCONS_00074002 and its target gene *colec10* suggests an activation of the complement system in the spleen to bolster the host immune response against *T. canis* invasion during the initial phase of *T. canis* infection.

At 96 hpi, 729 DEmRNAs were significantly enriched in 494 GO terms (Additional file 8: Table S6), of which 23 GO terms were related to immune response and inflammation, and 181 DEmRNAs were significantly enriched in 11 signaling pathways (Additional file 10: Table S7). *ifit1* was significantly enriched in 41 GO terms, such as innate immune response, regulation of immune effector process and response to type I interferon. *ifit2* was significantly enriched in 14 GO terms, such as response to INF-α, defense response and immune effector process. Unlike the level of *ifit1* which was downregulated 2.83-fold at 24 hpi, the transcriptional level of *ifit1* was upregulated 2.41-fold at 96 hpi, while the level of TCONS_00345683 located near the *ifit1* gene was downregulated 9133.32-fold.

The IFN-*γ* signaling and TLR signaling pathways associated with *ifit1* were activated. *ifit2* belongs to the IFIT gene family and could be regulated by the same lncRNA because *ifit1* was also upregulated 2.15 fold at 96 hpi. The pathways associated with *ifit2* are IFN-*γ* signaling and the immune response IFN-α/-*β* signaling pathway [46]. There are many cellular responses that can counter infection, including the secretion of INFs [46]. Transmembrane and cell membrane pattern recognition receptors (PRRs) that recognize microbial nucleic acids and other macromolecules activate signaling pathways that induce INFs, which leads to transcriptional induction of nuclear genes [47]. The INF-stimulated genes (ISGs), such as ISG54, together with *ifit2* form complexes that can regulate various cellular responses to INF or infection [48]. The ISG54/*ifit2* complex is induced as a primary stress response to infection, which is a primary response to IFNs and PRRs and mediates mitochondrial-related cellular apoptosis [49]. Ectopic expression of ISG54/*ifit2* promotes death of the transformed cells [46]. In the present study, *T. canis* infection in Beagle puppies led to upregulation of *ifit2*, which could enhance stress response to infection or mediate the death of infected host cells to suppress *T. canis* infection.

Protein tyrosine phosphatase non-receptor type 14 (*ptpn14*) was significantly enriched in five GO terms, such as cytoplasm, protein metabolic process and cellular protein metabolic process. *Ptpn14* is a unique regulator of the mammalian lymphatic development and human biliary tract development and may be involved in regulating lymphangiogenesis [50]. At 96 hpi, the expression level of *ptpn14* was increased 3.22-fold, and the level of TCONS_00408594 located near the *ptpn14* gene was downregulated 255.64 fold. The upregulation of *ptpn14* expression and the downregulation of TCONS_00408594 expression may promote lymphatic vessel development, enhancing the immune function in infected dogs.

WAS/WASL interacting protein family member 1 (*wipf1*) encodes WAS/WASL-interacting protein (WIP) and was significantly enriched in six GO terms at 96 hpi, such as response to other organisms, multi-organism process and response to biotic stimulus. The absence of WIP can affect adaptive immune cells, including B and T cells; WIP-deficient patients suffer from T-cell lymphopenia, especially CD8^+^ T cells [51]. In the absence of WIP, cytotoxicity can be mediated by reduction of natural killer (NK) cells and chemotaxis of B cells; however, when WIP expression is restored, these cellular alterations can be rescued [52]. In addition, WIP has been identified as an MKL (Megakaryocytic leukemia)-dependent serum response factor (SRF)-target gene [53]. MKL1 contributes to the maintenance of immune cells, which may affect the function and migration of myeloid cells [52]. At 96 hpi, the level of *wipf1* gene was upregulated 1.52-fold, and the level of TCONS_00332139, located near the *wipf1* gene, was downregulated 1403.09-fold. The alteration of *wipf1* expression may influence the function of adaptive immune cells, such as B and T cells.

At 36 dpi, the results of GO and KEGG analyses showed that 350 DEmRNAs were significantly enriched in 416 GO terms (Additional file 8: Table S6). Of these, 14 GO terms were associated with immune response or inflammation, and 67 DEmRNAs were enriched in 14 signaling pathways (Additional file 10: Table S7). *Ube2s* was significantly enriched in eight GO terms, such as regulation of ubiquitin homeostasis, free ubiquitin chain polymerization and ubiquitin homeostasis. *Ube2s* is the negative regulator of type I IFN signaling, which interacts with and attenuates TBK1 polyubiquitination and TANK-binding kinase 1 (TBK1) to remove the Lys63 (K63)-linked polyubiquitin of TBK1 by combining the ubiquitin-specific protease 15 (USP15), thereby inhibiting type I IFN production [54]. This suggests that the *ube2s*-USP15-TBK1 axis contributes to maintaining the innate immune response against infection [55]. At 36 dpi, the transcriptional level of *ube2s* was downregulated 1.63-fold, and the transcriptional level of TCONS_00023346, located near the *ube2s* gene was downregulated 785.13-fold. The downregulation of *ube2s* expression may increase IFN production and improves host immunity against infection.

Taken together, the patterns of DElncRNAs and DEmRNAs identified in the present study indicate that host immune response increases at 96 hpi when *T. canis* is present in the lungs of Beagle puppies, while at 36 dpi, host immune activity becomes slightly suppressed when *T. canis* has completed its tracheal migration and settled in the intestine. These findings are in agreement with previous results showing that the development of *T. canis* in the definitive canine host is mediated by a dynamic balance between pro-inflammatory and anti-inflammatory responses [10].

## Conclusions

Using next-generation sequencing, we have performed, for the first time, a quantitative and comprehensive analysis of the noncoding and coding transcriptomes of the spleen of Beagle puppies infected by *T. canis*. The analysis revealed significant differences in the abundance and expression patterns of lncRNAs and mRNAs in the spleen from infected and uninfected puppies, as well as dynamic transcriptional changes as infection advances. Our results suggest that the expression patterns of distinct lncRNAs and mRNAs in the spleen were *T. canis* infection-specific and responsive to the progression of infection. Exploring the functional roles of these lncRNAs and mRNAs should provide more mechanistic insights into the pathophysiology of toxocariasis.

## Supplementary Information

**Additional file 1: Table S1**. The primers used in the qRT-PCR experiment.

**Additional file 2: Table S2**. The differentially expressed lncRNAs and mRNAs at 24 hpi, 96 hpi and 36 dpi.

**Additional file 3: Figure S1**. Co-localization between the differentially expressed (DE) lncRNA transcripts and their potential target DEmRNAs at (**a**) 24 h post-infection (hpi), (**b**) 96 hpi and (**c**) 36 days post-infection (dpi). Red and green colors represent upregulated and downregulated DElncRNA transcripts and DEmRNAs, respectively. The ellipses represent DEmRNAs and diamonds represent DElncRNA transcripts.

**Additional file 4: Table S3**. Co-localization between the differentially expressed lncRNA transcripts and their predicted differentially expressed *cis* target genes.

**Additional file 5: Table S4**. Gene Ontology (GO) terms of *cis* target genes of the differentially expressed lncRNAs.

**Additional file 6: Figure S2**. Scatter plots of the top 20 enriched Kyoto Encyclopedia of Genes and Genomes (KEGG) pathways of the dysregulated lncRNA transcript targeted genes at (**a**) 24 h post-infection (hpi), (**b**) 96 hpi, and (**c**) 36 days post-infection (dpi) in the spleen of Beagle dogs. The X-axis label represents the rich factor; the Y-axis label shows the KEGG pathways. The rich factor reflects the proportion of lncRNA transcript targeted genes in a given pathway. The color of the dots represents the enrichment score [− log_10_ (*P*-value)], where red color indicates high enrichment, while green color indicates low enrichment. Dot size represents the number of lncRNA transcript targeted genes in the corresponding pathway (bigger dots indicate large lncRNA transcript targeted genes number).

**Additional file 7: Table S5**. The KEGG pathways of *cis* target genes of the differentially expressed lncRNA transcripts.

**Additional file 8: Table S6**. Gene Ontology (GO) terms of the differentially expressed mRNAs.

**Additional file 9: Figure S3**. Scatter plots of the top 30 enriched Gene Ontology (GO) terms (including biological process, cellular component and molecular function categories) of the DEmRNAs at (**a**) 24 h post-infection (hpi), (**b**) 96 hpi, and (**c**) 36 days post-infection (dpi) in the spleen of Beagle dogs. The X-axis label represents the rich factor; the Y-axis label shows the GO terms. The rich factor reflects the proportion of DEmRNAs in a given GO term. The color of the dots represents the enrichment score [− log_10_ (*P*-value)], where red color indicates high enrichment, while green color indicates low enrichment. Dot size represents the number of DEmRNAs in the respective GO term (bigger dots indicate large DEmRNA number). AMPA, Alpha-amino-3-hydroxy-5-methyl-4-isoxazolepropionic acid; CCIC, cytosolic calcium ion concentration; PCGC, phospholipase C-activating G-protein coupled

**Additional file 10: Table S7**. The KEGG pathways of the differentially expressed mRNAs.

## Data Availability

The datasets supporting the findings of this article are included within the paper and its supplementary materials. The RNA-seq raw data described in the present study has been submitted to the NCBI Short Read Archive database (https://www.ncbi.nlm.nih.gov/sra) under the bio-project number PRJNA789949.

## Abbreviations

CL-L1: Lectin liver 1
CL-11: Collectin kidney 1
*colec10*: Collectin subfamily member 10
DElncRNA transcripts: Differentially expressed lncRNA transcripts
DEmRNAs: Differentially expressed mRNAs
dpi: Days post-infection
GO: Gene Ontology
hpi: Hours post-infection
IFIT: Interferon-inducible protein
*ifit1*: Interferon-induced protein with tetratricopeptide repeats 1
IFN: Interferon
ISGs: IFN-stimulated genes
K63: Lys63
KEGG: Kyoto Encyclopedia of Genes and Genomes
L13A: Ribosomal protein L13A
lncRNA: Long non-coding RNA
MKL: Megakaryocytic leukemia
mRNA: Messenger RNA
NK: Natural killer
NT: Neurotoxocariasis
OLM: Ocular larva migrans
*prkx*: Protein kinase X-linked
PRRs: Pattern recognition receptors
*ptpn14*: Protein tyrosine phosphatase non-receptor type 14
qRT-PCR: Quantitative real-time PCR
RIN: RNA integrity number
RNA-seq: RNA sequencing
*rorc*: Retinoic acid receptor (RAR)-associated orphan receptor C
SRF: Serum response factor
TBK1: TANK-binding kinase 1
*tcf7l2*: Transcription factor 7 like 2
TCF/LEF: T-cell factor/lymphatic enhancer factor
*tnfrsf11a*: TNF receptor superfamily member 11a
*ube2s*: Ubiquitin-conjugating enzyme E2S
USP15: Ubiquitin-specific protease 15
VLM: Visceral larva migrans
*wipf1*: WAS/WASL interacting protein family member 1
