# Supplementary figures and images for: RNA sequencing reveals dynamic expression of spleen lncRNAs and mRNAs in Beagle dogs infected by Toxocara canis
Source: Parasit Vectors. 2022 Aug 4;15:279. doi: 10.1186/s13071-022-05380-x (PMC9351231; doi:10.1186/s13071-022-05380-x)

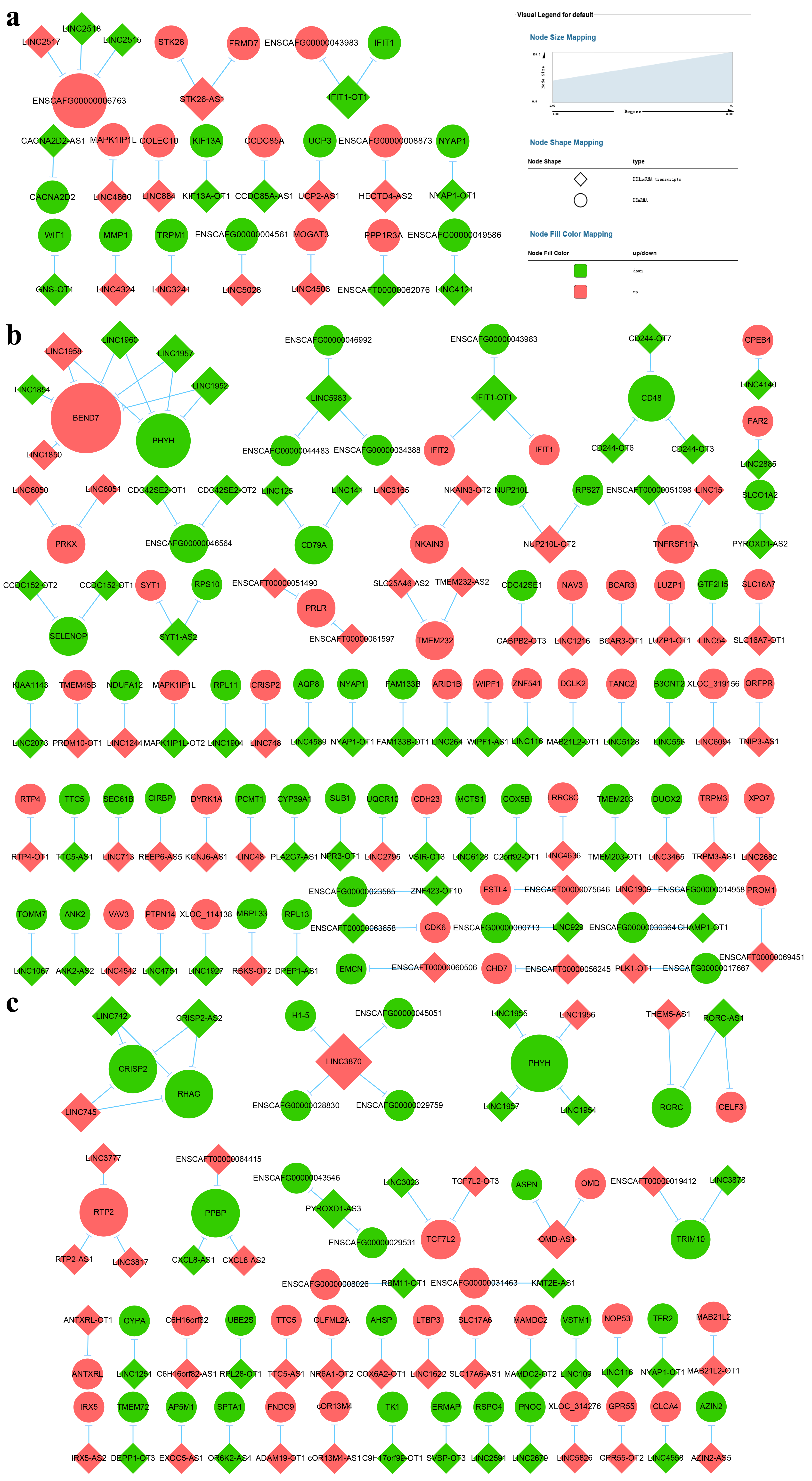

Supplement: Supplementary file 3 — Additional file 3: Figure S1. Co-localization between the differentially expressed (DE) lncRNA transcripts and their potential target DEmRNAs at (a) 24 h post-infection (hpi), (b) 96 hpi and (c) 36 days post-infection (dpi). Red and green colors represent upregulated and downregulated DElncRNA transcripts and DEmRNAs, respectively. The ellipses represent DEmRNAs and diamonds represent DElncRNA transcripts. [file 13071_2022_5380_MOESM3_ESM.tif]

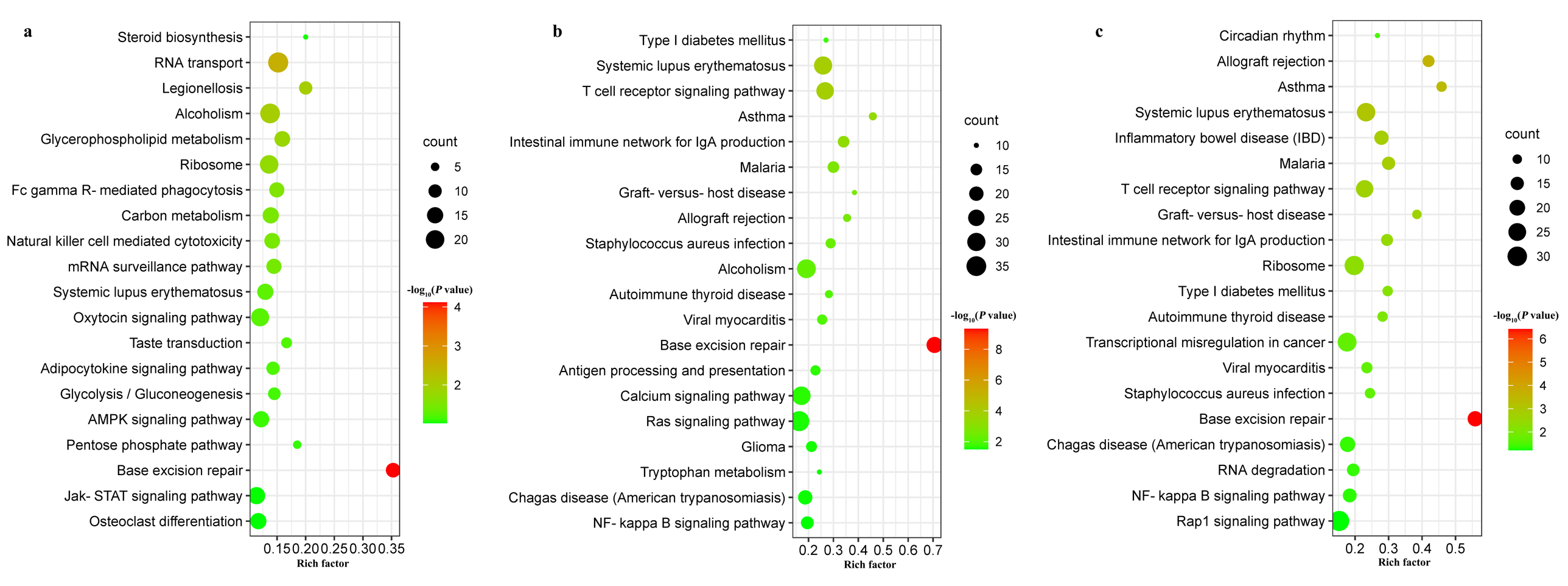

Supplement: Supplementary file 6 — Additional file 6: Figure S2. Scatter plots of the top 20 enriched Kyoto Encyclopedia of Genes and Genomes (KEGG) pathways of the dysregulated lncRNA transcript targeted genes at (a) 24 h post-infection (hpi), (b) 96 hpi, and (c) 36 days post-infection (dpi) in the spleen of Beagle dogs. The X-axis label represents the rich factor; the Y-axis label shows the KEGG pathways. The rich factor reflects the proportion of lncRNA transcript targeted genes in a given pathway. The color of the dots represents the enrichment score [− log10 (P-value)], where red color indicates high enrichment, while green color indicates low enrichment. Dot size represents the number of lncRNA transcript targeted genes in the corresponding pathway (bigger dots indicate large lncRNA transcript targeted genes number). [file 13071_2022_5380_MOESM6_ESM.tif]

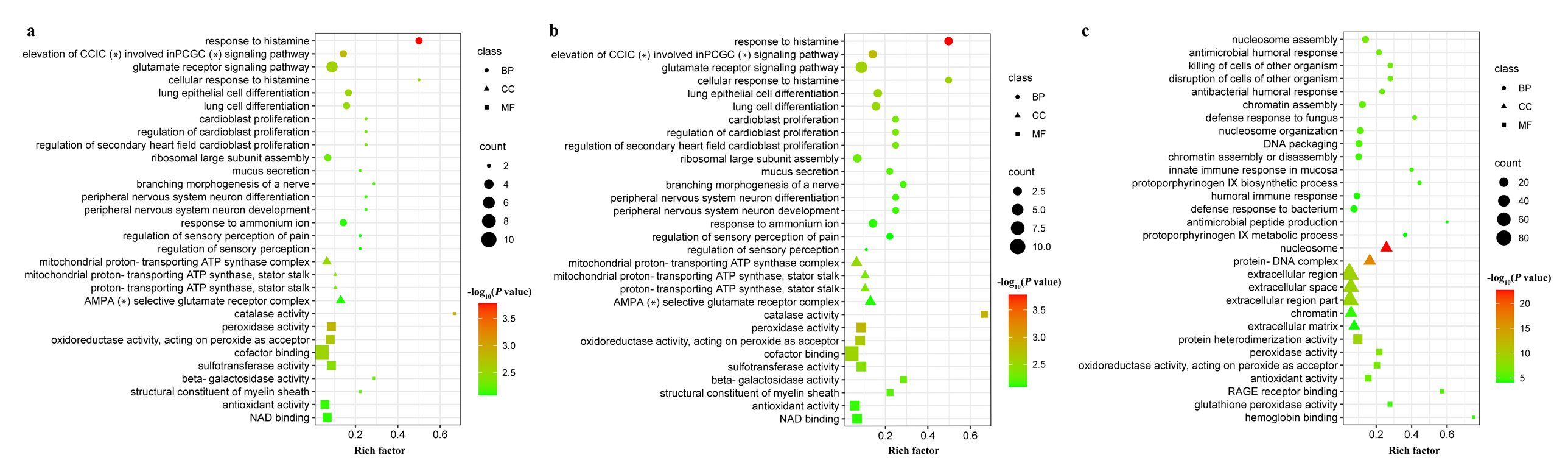

Supplement: Supplementary file 9 — Additional file 9: Figure S3. Scatter plots of the top 30 enriched Gene Ontology (GO) terms (including biological process, cellular component and molecular function categories) of the DEmRNAs at (a) 24 h post-infection (hpi), (b) 96 hpi, and (c) 36 days post-infection (dpi) in the spleen of Beagle dogs. The X-axis label represents the rich factor; the Y-axis label shows the GO terms. The rich factor reflects the proportion of DEmRNAs in a given GO term. The color of the dots represents the enrichment score [− log10 (P-value)], where red color indicates high enrichment, while green color indicates low enrichment. Dot size represents the number of DEmRNAs in the respective GO term (bigger dots indicate large DEmRNA number). AMPA, Alpha-amino-3-hydroxy-5-methyl-4-isoxazolepropionic acid; CCIC, cytosolic calcium ion concentration; PCGC, phospholipase C-activating G-protein coupled [file 13071_2022_5380_MOESM9_ESM.tif]
